# South West, Wessex and South Wales Rheumatology Club

**Published:** 1983-04

**Authors:** 


### South West, Wessex and South Wales Rheumatology Club

Abstracts of Papers given at the meeting held at Bristol Royal Infirmary on 10th December 1982

### LEG ULCERS IN RHEUMATOID ARTHRITIS

**O. A. Thurtle, A. G. MacIvor and M. I. D. Cawley, Departments of Rheumatology and Pathology, Southampton**

We shall soon be reporting a twofold increase in leg ulceration in rheumatoid arthritis (RA). Although frequently attributed to vasculitis the precise pathogenesis is uncertain. For 2 years we studied prospectively all 15 patients with RA complicated by leg ulceration who presented to us to search for possible causes.

We noted the clinical appearance of the ulcers and looked for various other factors thought to contribute to ulcer formation. We also performed a biopsy of the ulcer and/or the rectum in nine patients.

Clinical appearance, solitary malleolar or lower calf eight, other ulcers seven.

The solitary malleolar ulcers looked venous. Only one had severe varicose veins but all the others had marked ankle involvement, severe in six. Only two of 15 matched controls had severe ankle involvement.

Of the seven patients with other ulcers, one was ischaemic, one the result of steroid atrophy, one had clinical vasculitis and of the remaining four one had skin biopsy evidence of vasulitis, two showed some features of pyoderma.

The two patients with vasculitis both had normal rectal biopsies as had the five patients with venous ulcers who were biopsied.

Solitary malleolar ulcers are probably venous, the result of ankle dysfunction.

In the non venous ulcers there was some evidence of vasculitis in all but two, both of whom had likely alternative causes. The evidence for vasculitis was strong in only two, other causes could be implicated in the three remaining patients.

### JOINT DISEASE IN SCLERODERMA

**L. J. Catoggio, G. Evison, J. A. L. Harkness and P. J. Maddison, Royal National Hospital for Rheumatic Diseases, Bath**

Arthritis in systemic sclerosis (SS) can be prominent and an overlap with rheumatoid arthritis (RA) has sometimes been queried. We therefore analysed the pattern of joint involvement of hands and wrists in 34 patients (one male) with SS and compared it with that of nine patients with mixed connective tissue disease (MCTD), a condition with distinct overlap features.

Although MCTD patients were younger, disease duration was similar in both groups. Extra articular radiological findings such as calcinosis, pulp atrophy and tuft resporption were present in over half the patients with SS but were less frequent in MCTD.

Marginal erosions occurred in over 50% and ulnar styloid erosions in 35% of the SS patients. Erosions of the 1st cmc joint were seen in one-third of them and were unrelated to age. Erosions did not correlate with clinical synovitis, the presence of rheumatoid factor which was present in 26% of SS patients or the presence of anti centromere or anti Scl-70 antibodies.

Erosions in MCTD patients occurred frequently and were more destructive and rapidly progressive. Correlation between erosions, rheumatoid factor and clinical arthritis was high.

We conclude that erosions in SS are frequent. Their pattern is varied but usually discrete and seldom destructive. They are not related to arthritis or serological abnormalities and may be due to several mechanisms aside from synovitis. In contrast, erosions in MCTD are more frequently destructive and related to arthritis and rheumatoid factor, suggesting a true overlap.

### RHEUMATIC SYMPTOMS IN PRIMARY HYPERPARATHYROIDISM

**M. Helliwell, Salisbury General Hospital**

In a retrospective survey of 34 patients with primary hyperparathyroidism (HPT), 18 (53%) complained of musculo-skeletal symptoms during the 12 months before the diagnosis was made and nine (26%) attended at some time for either a rheumatological or orthopaedic consultation. Myalgia was the most frequently reported symptom which occurred in 41% of patients. Arthralgia, mainly affecting the large joints was present in 11 (32%) patients, two of whom had an erosive synovitis mimicking rheumatoid arthritis. Radiological abnormalities were seen in eight patients; these findings are described. Clinicians should be aware of the variety and frequency of musculo-skeletal symptoms associated with HPT and should consider including serum calcium measurements when investigating rheumatic complaints.

### SPONDYLOARTHRITIS IN BEHÇET'S SYNDROME

**E. R. Higgs, N. Caporn, P. Dieppe and I. Watt, Bristol**

In Behçet's syndrome, peripheral arthropathy is a well-recognised feature, but there are conflicting reports of the prevalence of sacro-iliitis.

We surveyed all definite cases of Behçet's syndrome in our Hospital Diagnostic Index. There were 14 patients, nine female and five male, with mean age of onset 29 years (range 3–55 years and mean duration of disease 14 years (range 3–38 years).

Thirteen patients had peripheral joint symptoms but only two had abnormalities on examination (knee effusions). None had radiological changes in the peripheral joints.

Ten patients had suffered low back pain, and six had sacro-iliac tenderness on examination (one bilaterally), nine patients showed radiological abnormalities of the sacro-iliac joints: erosive changes (seven patients), subarticular sclerosis (seven patients) and joint-space narrowing (four patients). Changes were bilateral in five patients, but symmetrical in only one: in one there was partial ankylosis. Six patients had minor degenerative changes of the lumbar spine, and one had vertebral body squaring. Five patients had calcaneal spurs, with features of an active erosive enthesopathy in two.

No patient was positive for the HLA B27 haplotype.

We conclude that sacro-iliitis is common in Behçet's syndrome. It is mild, and usually asymmetrical. The lack of association with HLA B27 suggests that Behçet's syndrome should not be grouped with other seronegative spondarthritides.

